# Structural Interface Forms and Their Involvement in Stabilization of Multidomain Proteins or Protein Complexes

**DOI:** 10.3390/ijms17101741

**Published:** 2016-10-18

**Authors:** Jacek Dygut, Barbara Kalinowska, Mateusz Banach, Monika Piwowar, Leszek Konieczny, Irena Roterman

**Affiliations:** 1 Department of Rehabilitation, Hospital in Przemyśl, Monte Cassino 18, 37-700 Przemyśl, Poland; jacekdygut@gmail.com; 2 Faculty of Physics, Astronomy and Applied Computer Science, Jagiellonian University, Łojasiewicza 11, 30-348 Krakow, Poland; malijka@gmail.com; 3 Department of Bioinformatics and Telemedicine, Jagiellonian University–Medical College, Łazarza 16, 31-530 Krakow, Poland; mateusz.banach@uj.edu.pl (M.B.); mpiwowar@cm-uj.krakow.pl (M.P.); 4 Chair of Medical Biochemistry, Jagiellonian University–Medical College, Kopernika 7, 31-034 Krakow, Poland; mbkoniec@cyf-kr.edu.pl

**Keywords:** hydrophobic core, homodimers, utrophin, dystrophin, domain, hydrophobicity, interface

## Abstract

The presented analysis concerns the inter-domain and inter-protein interface in protein complexes. We propose extending the traditional understanding of the protein domain as a function of local compactness with an additional criterion which refers to the presence of a well-defined hydrophobic core. Interface areas in selected homodimers vary with respect to their contribution to share as well as individual (domain-specific) hydrophobic cores. The basic definition of a protein domain, i.e., a structural unit characterized by tighter packing than its immediate environment, is extended in order to acknowledge the role of a structured hydrophobic core, which includes the interface area. The hydrophobic properties of interfaces vary depending on the status of interacting domains—In this context we can distinguish: (1) Shared hydrophobic cores (spanning the whole dimer); (2) Individual hydrophobic cores present in each monomer irrespective of whether the dimer contains a shared core. Analysis of interfaces in dystrophin and utrophin indicates the presence of an additional quasi-domain with a prominent hydrophobic core, consisting of fragments contributed by both monomers. In addition, we have also attempted to determine the relationship between the type of interface (as categorized above) and the biological function of each complex. This analysis is entirely based on the fuzzy oil drop model.

## 1. Introduction

The traditional definition of a domain refers to the local compactness criterion [1,2,3,4]. Accordingly, the domain is regarded as an independent entity, which may exist in separation from the rest of the polypeptide chain [5,6]. This notion is supported by the fact that domains often evolve independently of one another. Residues responsible for mediating the protein’s biological function may be restricted to a single domain or spread across multiple domains.

Two databases—SCOP [7,8] and CATH [9]—Group proteins with respect to their sequential and structural similarity, introduce suitable classifications for both purposes. The sequential similarity factor highlights evolutionary changes which produce homologues. From an evolutionary point of view, changes in the protein’s function may call for individual structural rearrangements in each domain. Given that domains often form functional units, their structural stability should be viewed as a critical factor.

Domain-specific structural changes are often discussed in the context of amyloidogenesis where partial unfolding of domain fragments (e.g., individual loops) is seen as a possible cause of amyloid plaque formation [10]. According to the domain swapping hypothesis a multimolecular aggregate emerges as a result of uncoiling of a domain fragment, leaving behind a pocket which accommodates the uncoiled fragment of another molecule [11]. Improperly folded proteins (or individual domains) lead to pathological changes commonly referred to as misfolding diseases [12].

Voronoi tessellation is a specialized model used in the study of inter-domain interfaces. [13,14,15]. Such interfaces are recognized as highly specific to their interaction partners and binding orientation. The relative positions of interface residues are generally well conserved within the same type of interface, even between remote homologues [16].

The relation between interface structure and hydrophobicity density distribution is particularly relevant in the context of amyloidogenesis [17]. Selective cross-saturation effects have been applied in docking method analysis [18].

A thorough discussion of protein complexation and interface conformation would not be possible without referring to the worldwide discussion forum offered by the CAPRI (Critical Assessment of PRedicted Interactions) project, originally launched in 2001 [19,20]. The goal of CAPRI is to predict the structure of protein complexes on the basis of the structural properties of individual monomers. After more than 10 years of development it seems that rigid body docking yields better results than the alternative flexible docking method, which calls for substantial conformational changes in target monomers. Successive editions of CAPRI introduce new challenges—For instance, assessment of the expected stability of protein complexes. Expectations related to the prediction of structural properties of complexes include determining their biological role, or—When this proves impossible—Identifying relations between the model and the biological activity of a specific complex [21]. It appears that chemical complementarity and conformational similarity are the main forces driving the formation of protein complexes.

A cursory review of techniques employed in the CAPRI challenge indicates one common characteristic: They all tend to focus on pairwise (atom-atom) interactions. Analysis of the protein-protein interface is usually restricted to the zone of contact, with the geometry of solids (monomers) often used as the sole criterion of structural compatibility.

In our work, we propose an approach which regards complexation as an effect of global conformational characteristics produced by the fuzzy oil drop model (FOD) in each structural unit (domain or protein), as well as in multi-domain complexes and protein dimers (note that we restrict our analysis to homodimers). The presence of a shared hydrophobic core is seen as the primary factor responsible for quaternary structural stabilization. The paper presents a set of homodimers in which the status of the inter-domain (or inter-chain) interface varies. Some dimers exhibit a shared hydrophobic core while in others no such core is present. In some cases we can attribute this phenomenon to the specific biological function of a given homodimer. Special attention is given to two proteins: dystrophin [22,23,24,25] and utrophin [26,27,28,29,30,31,32,33].

All homodimers presented in this work have been analyzed with the use of the fuzzy oil drop model [34]—An extension of the original oil drop model [35], which predicted internalization of hydrophobic residues along with exposure of hydrophilic residues on the surface. This qualitative concept has been extended with quantitative analysis by introduction of a mathematical model in which the “expected” hydrophobicity density distribution is represented by a Gaussian defined over a 3D ellipsoid and peaking at the geometric center of the molecule. This “idealized” distribution of hydrophobicity density is then compared with the actual (observed) distribution, which depends on the placement and properties of each residue in the protein structure. Such comparison enables us to identify locally discordant areas. The accuracy of the fuzzy oil drop model is confirmed by the study of protein families in which biological activity requires close correspondence between the observed and theoretical distributions (e.g., antifreeze and downhill proteins) [36,37]. Locally significant deviations from the idealized model indicate possible areas of interaction [38], with ligand binding pockets and protein complexation sites traceable to local hydrophobicity deficiencies and excesses respectively [39,40,41].

The innovation of the fuzzy oil drop approach rests in its ability to identify “hydrophobically ordered” domains in the protein body—Even when these domains are actually formed by fragments contributed by separate chains. Such emergent hydrophobic cores may play a key role in ensuring proper biological activity of protein complexes, particularly when contraction and stretching come into play [42]. The presence of a hydrophobic core appears to be a significant factor in quaternary stabilization of protein complexes [43].

To-date analyses of homodimers on the basis of the fuzzy oil drop model characterize protein-protein interfaces as areas exhibiting higher-than-expected hydrophobicity—A result of local exposure of hydrophobic residues on the protein surface [39,40,41]. A detailed study of homodimers suggests that both the dimer and its constituent monomers may vary with respect to their adherence to the fuzzy oil drop model (FOD model). A well-ordered hydrophobic core may be present in the dimer as well as in each monomer separately. In the former case the core is shared by both interacting monomers, contributing to structural stabilization of the complex.

In light of the above, we can divide homodimers into four distinct groups: (1) hydrophobic core present in each participating monomer as well as in the dimer; (2) hydrophobic core present in the dimer but not in either monomer; (3) hydrophobic core present in both monomers but not in the dimer; and (4) hydrophobic core present in one participating monomer as well as in the dimer. The main point of this paper is the recognition of a distinct interface which manifests itself as a quasi-domain comprising fragments contributed by both monomers and possessing its own hydrophobic core. Such quasi-domains have been found in dystrophin and utrophin.

The analysis presented in this work acknowledges the FOD status of homodimers (as explained above), focusing on representative cases for each group.

The presented homodimers have been selected in such a way as to cover the entire spectrum of relationships between individual units (domains, chains) (Table 1):

(1) Hydrophobic core (according to FOD criterion) present in each participating monomer as well as in the dimer. This case is represented by the histone B dimer from *Methanothermus fervidus* (a hyperthermophilic organism)—1BFM [44].

(2) Hydrophobic core present (according to FOD model) in the dimer but not present in either monomer, represented by protein 1Y7Q [45]. 1Y7Q is a mammalian SCAN domain dimer which is a domain-swapped homologue of the HIV capsid C-terminal domain. The structure of 1Y7Q is interesting as it adopts a fold almost identical to that of the retroviral capsid (CTD) but uses an entirely different dimerization interface produced by swapping the MHR-like element between monomers.

(3) Hydrophobic core present in both monomers but not in the dimer; the metal-sensing transcriptional repressor from *Staphylococcus aureus* in the Zn2-form (1R1V) is representative of this group [46].

(4) Hydrophobic core present in one participating monomer as well as in the dimer. This category is represented by the 2CD0—Human lambda-6 light chain dimer, a variable domain from the lambda-6 type immunoglobulin light chain [47]. This dimer may rise doubts due to the dissymmetry observed in homodimer (usually the C2 symmetry is expected in this case) and due to the high value of Rfree = 0.351. This protein is occurring in pathological conditions (over-expression of immunoglobulin light chain) and sometimes undergoes the amyloid transformation [47]. The observed structure, however related to pathological conditions, is evidenced and additionally may code some aspects allowing amyloid transformation [48].

The main contribution of this paper is recognition of a distinct interface, which manifests itself as an independent domain, comprising fragments contributed by both monomers and possessing its own well-defined hydrophobic core. Such interfaces are found in dystrophin and utrophin.

Dystrophin is one of the largest proteins found in humans. It is expressed in various tissues, including skeletal muscle, heart, nerves and liver, where it forms an important component of the cellular membrane. Dystrophin is a cytoplasmic protein with an elongated shape. It links the cytoskeleton of the muscle cell with the extracellular matrix across the plasma membrane [48]. Dystrophin is coded for by the DMD gene located at Xp21.2-p21.1 and consisting of 79 exons [23,24]. Mutations in DMD result in synthesis of an aberrant protein which, in turn, causes progressive muscular dystrophy. In its native form, dystrophin is a rodlike molecule, approximately 150 nm long, located beneath the cellular membrane. The transcript consists of 3685 amino acid residues (NP_03997.1) forming four domains which mediate interaction with cytoskeletal proteins (actin filaments) on the N-terminal side, and with membrane glycoproteins on the C-terminal side (dystrophin-associated proteins (DAPs) and dystrophin-associated glycoproteins (DAGs) respectively). Our analysis is restricted to the N-terminal actin-binding domain of human dystrophin containing CH1 and CH2 domains, present in each of the two chains which make up the homodimer. The structure of dystrophin (PDB: 1DXX) is listed as consisting of four chains paired up to form two subunits with identical symmetry (AB + CD). Our analysis focuses on the AB dimer, which is identical to the CD dimer.

Utrophin [25] is another large molecule found in the cellular membrane and expressed with particular intensity during embryonic development, as well as in regenerating muscle tissue. Utrophin (1QAG) is a cytoskeletal protein homologous to dystrophin. Its degree of sequential similarity reaches 85% in acting-binding domains. Utrophin is responsible for mediating the activity of muscle cells to which it contributes by stabilizing plasmatic membranes. It is also responsible for clustering of the acetylcholine receptor. Dystrophin and utrophin expression is greatly upregulated in patients suffering from Duchenne muscular dystrophy [24]. The PDB structure (1QAG [25]) comprises two chains arranged in a similar way to the AB complex of dystrophin. This fragment is also responsible for binding to actin. Utrophin is generally undetectable in healthy adults. When expressed, it can be found at the neuromuscular synapse and myotendinous junctions. In all other locations it is replaced by dystrophin, which it structurally resembles. Utrophin is coded for by the UTRN gene, consisting of 74 exons and located at 6q24 [27]. The transcript consists of 3433 residues (NP_009055.2). Its N-terminal fragment interacts with actin while the C-terminal fragment is responsible for binding to AChR in the neuromuscular synapse [28,29].

Both utrophin and dystrophin interact with actin in a similar way. They both affect the mobility of actin—Specifically, its bending and twisting capabilities [30]. Functional differences between both proteins relate mostly to their site of interaction with actin. Dystrophin attaches to actin in two low-affinity areas while utrophin makes use of a higher-affinity fragment (continuous actin-binding domain). As a result, the torsional rigidity of actin is more significantly impaired by dystrophin than by utrophin. It is believed that the interaction between actin and dystrophin (or utrophin) promotes stretching and twisting, determining the stability of actin filaments.

According to some studies either dystrophin or utrophin may have formed via partial gene duplication [31]. In biological nomenclature both proteins are referred to as paralogues, implying that they are homologous [32].

The set of proteins under consideration is presented in Table 1 with a brief description of the status of the hydrophobic core in each example.

### The Fuzzy Oil Drop Model: An Introduction

The fuzzy oil drop model is based on the oil drop introduced by Kauzmann assuming hydrophobic residues migrating to the center of the protein body with hydrophilic residues exposed on its surface [39,40,41]. The modification introduced in fuzzy oil drop model uses the 3D Gauss function to express the continuous decrease of hydrophobicity in the distance from the molecule center (maximum hydrophobicity) toward the surface where the hydrophobicity takes the level close to zero. The assumed idealized distribution of hydrophobicity compared with the hydrophobicity observed in the real molecule (inter-residual hydrophobic interaction) reveals the differences. They can be of global or local character. The quantitative measurement of these differences is possible applying the Kullback–Leibler entropy [49].

The global discordance expressed by RD > 0.5 for complete protein (or domain) is interpreted as lack of ordered hydrophobicity core in particular molecule. Local discordance is also expressed by RD > 0.5 calculated for selected fragment of the profiles (T-expected, O-observed). In particular, the fragments of polypeptide chain representing well defined secondary structure can be taken as units to calculate RD to characterize their status versus the ordered hydrophobic core. It is also possible to calculate the RD value for the polypeptide chain with selected residues eliminated from calculation. This is to show the role of certain (selected) residues in the hydrophobic core construction. In particular, the resides engaged in protein-protein interaction can be used as objects to be eliminated. Their status depends on the internal construction of the domain (molecule) and on the external factors which is the second protein molecule. The residues located in the inter-protein interface are the object of the analysis presented here. The aim of this analysis is the definition of the role of residues responsible for dimers construction and its stability.

To visualize the status of particular chain, domain or chain fragment the status of RD > 0.5 discordance versus the theoretical distribution expressed by the values of RD above 0.5 are given in bold.

The fuzzy oil drop model is described in details in a paper recently published [50]. This is why the description presented here is limited to the main points of the procedure.

Identification of secondary structural folds and the composition of protein domains follows the CATH and PDBSum classifications. Inter-domain/inter-chain contacts have been identified on the basis of the PDBSum distance criterion [51].

## 2. Results

### 2.1. Hydrophobic Core Present in the Dimer and in both Monomers

This category is represented by the histone B dimer from *Methanothermus fervidus* (a hyperthermophilic organism) labeled 1BFM [44].

Analysis of RD values listed in Table 2 indicates the presence of a shared dimeric core as well as individual monomeric cores. The dimeric core is more prominent than the monomeric cores. Analysis of individual helical folds reveals the destabilizing effect of the 21–51 fragment upon monomeric cores. Instead, the fragment appears to reinforce the shared core. The helical fold at 56–68 appears to play a significant role in shaping the monomeric cores as well as the shared dimeric core.

**Figure 1:**
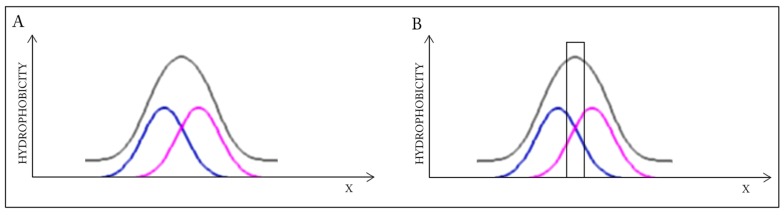
Schematic depiction of a system in which the dimer as well as its individual monomers contain well-defined hydrophobic cores. Elimination (black bar on the right profile) of residues involved in complexation distorts the dimeric core. Blue and pink lines: distributions in monomers, gray line: distribution in dimer. (**A**) Status of the individual monomers (**blue** and **pink**) versus the resultant distribution in dimer (**gray**); (**B**) The removal of the interface (**black bar**) does not change the status of the dimer—Distribution accordant versus the expected one.

Elimination of fragments, which participate in the inter-chain interface, distorts the shared core (RD increases from 0.383 to 0.390). This suggests that the interface zone contributes to the common hydrophobic core and its elimination destabilizes the complex, distorting the alignment between the observed and ideal distributions (Figure 1).

The presented protein comes from *Methanothermus fervidus*, an archaeon that grows optimally at 83 °C, making it a hyperthermophilic organism. It is, therefore, a very interesting study subject. Our experience indicates that well-ordered hydrophobic cores are particularly common in thermophilic proteins (publication currently in preparation). This is consistent with experimental studies which point to increased role of hydrophobic interactions under high temperature conditions [52].

A highly simplified, one-dimensional visualization of the presented case is shown in Figure 1. The figure reveals how two structures with accordant distributions merge to produce a coherent Gaussian which, by itself, also remains consistent with theoretical expectations. Elimination of interface residues in this particular case lowers the accordance of the dimer, as well as of its individual monomeric units.

### 2.2. Hydrophobic Core Present in the Dimer but Absent in both Monomers

This category is represented by 1Y7Q (Table 1). Fuzzy oil drop analysis indicates that the dimer possesses a shared hydrophobic core (RD = 0.421) while individual monomers lack such a core (RD = 0.553 and 0.514 respectively; Table 3). Parameters describing the status of each monomer and of the dimer itself suggest that individual secondary folds tend to participate in the shared core rather than in individual (monomeric) cores.

The interface zone plays a significant role in shaping the shared hydrophobic core: elimination of its residues (row labeled “No P-P”) increases the value of RD, which implies substantial involvement in construction of the shared core. We may conclude that this portion of the chain is the main component of the shared core and should be characterized by high local hydrophobicity. Reductions in RD calculated for individual secondary folds support this conclusion, suggesting that those fragments are aligned with the shared core.

Comparing RD values for both monomers and for the dimer reveals symmetry in the shared core, while individual assessment of each monomeric core points to certain differences—It seems that each monomer adapts to the shared core in a slightly different manner. We can conclude that most residues contribute to the shared core rather than to individual cores. From the point of view of a single monomer involvement of its residues in the shared (dimeric) core introduces deformations in the individual (monomeric) core.

The role of individual helical folds varies. Three helixes (four in the B chain) follow the expected monomeric distribution. When considering the dimer as a whole, four folds remain accordant with theoretical predictions. Of particular note is the helix at 60–76, which diverges from the idealized distribution in each monomer while exerting a strong stabilizing influence on the dimer (low RD values). This phenomenon can be explained by pointing out that the helix forms part of the interface zone and therefore plays a crucial part in quaternary stabilization.

From a functional point of view, the complex contains a zinc finger-associated SCAN domain, which is a homologue of the HIV-1 capsid protein. Hydrophobic core analysis may therefore provide a new model for protein-protein interactions underpinning viral particle formation [53].

**Figure 2:**
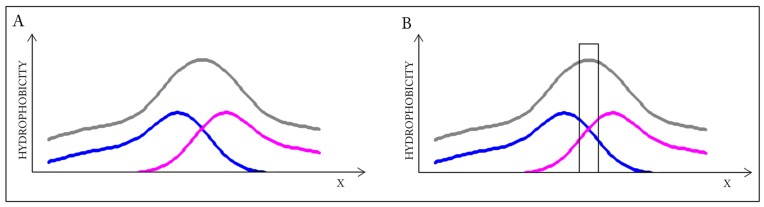
Schematic depiction of a system in which individual monomers lack hydrophobic cores but the dimer as a whole contains a well-defined core. Elimination of residues involved in complexation distorts the dimeric core while bolstering the monomeric cores—The black frame (right-hand schema) symbolizes the interface, which has been eliminated from RD calculation to characterize its influence on the status of the dimer, as well as the status of each monomer. Blue and pink lines: distributions in monomers, gray line: distribution in dimer. (**A**) Status of the individual monomers (**blue** and **pink**) versus the resultant distribution in dimer (**gray**); (**B**) the removal of the interface (**black bar**) does not change the status of the dimer—Distribution accordant versus the expected one.

This highly simplified, one-dimensional presentation of the presented case (Figure 2) reveals how two structures with divergent distributions produce a coherent Gaussian in close correspondence with theoretical predictions. The effect of elimination of interface residues is also depicted. It seems that in this case the interface plays a significant role in shaping the common (dimeric) core.

**Figure 3:**
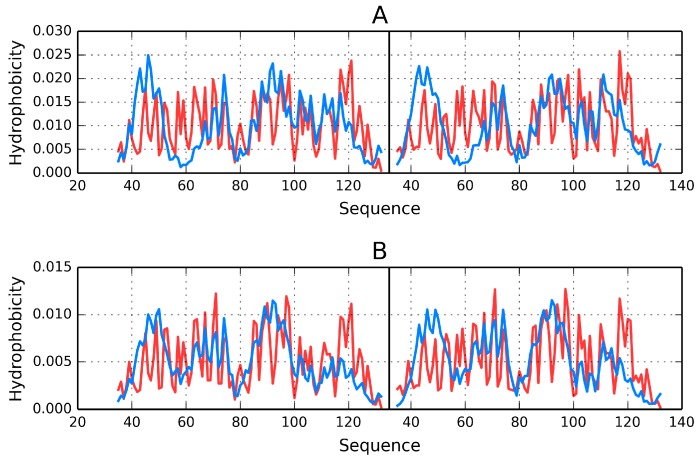
Hydrophobicity density distributions (theoretical: blue line; observed: red line) in 1Y7Q. (**A**) Monomer chain A and B respectively; (**B**) chain A and B in dimmer (common ellipsoid for both chains).

The charts shown in Figure 3 visualize the degree of accordance/discordance between theoretical and observed distributions for individual monomers and for the dimer. Poor accordance of monomeric distributions is particularly evident in the N-terminal portion of chain where the observed density is lower than expected (this relation reverses at around 50–70 aa). A different result is obtained when both monomers are regarded as components of a dimer.

### 2.3. Hydrophobic Core Absent in the Dimer but Present in both Monomers

This category is represented by the metal-sensing transcriptional repressor from *Staphylococcus aureus* in the Zn2-form (1R1V).

The dimer lacks a shared core while each monomer retains a well-defined core. The status of β-sheets both in the dimer and in each monomer separately indicates good accordance with the theoretical model. We can therefore conclude that β-sheets contribute to structural stability of both the dimer and each monomer.

Most secondary folds in 1R1V appear to stabilize their parent monomer rather than the protein-protein complex. This is particularly true of the helixes at 25–38 and 41–50 (Table 4).

Some other folds (such as the β-fragment at 68–74) play an equal role in the stabilization of monomeric units and the dimer. Elimination of interface residues improves the dimer’s accordance, suggesting that, at least in the case of 1R1V, protein complexation is mediated by forces other than hydrophobic interactions. Further analysis indicates that strong electrostatic forces are at play since the interface is composed almost entirely of polar residues.

The helixes at 52–66 and 84–100 do not align with the hydrophobic core; instead, they are involved in binding ligands (Zn^2+^ ions), which is critical for the protein’s biological function. The labile nature of both helixes is related to the required conformational changes when interacting with DNA strains [46].

**Figure 4:**
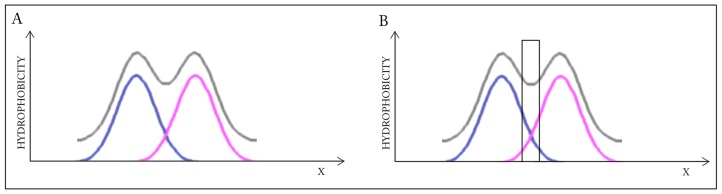
Schematic depiction of a system in which the dimer lacks a clear hydrophobic core while each monomer retains a well-defined core. Elimination of residues involved in complexation (right-hand image) leads to better accordance with the model when considering the dimer as a whole. Blue and pink lines: distributions in monomers, gray line: distribution in dimer. (**A**) Status of the individual monomers (**blue** and **pink**) versus the resultant distribution in dimer (**gray**); (**B**) Removal of the interface (**black bar**) changes the status of the dimer—Distribution appears accordant versus the expected one.

Figure 4 presents a model in which each monomer conforms to the idealized Gaussian while the dimer as a whole diverges from theoretical predictions. Elimination of interface residues improves the accordance of the dimer.

### 2.4. Hydrophobic Core Absent in the Dimer but Present in a Single Monomer

This category is represented by 2CD0: human lambda-6 light chain dimer (Bence-Jones protein), a variable domain from the lambda-6 type immunoglobulin light chain [47].

The protein is a homodimer consisting of two V immunoglobulin domains. Despite sharing a β-sandwich structure, both domains differ with respect to their FOD status. The dimer itself diverges from the model as does one of its monomeric subunits. Nevertheless, individual secondary folds appear to play a similar role in the stabilization of each monomer; for example, the upper β-sheet (called the upper core) is a good match for theoretical predictions in the dimer as well as in both monomers, while the lower β-sheet (lower core) significantly diverges from the idealized distribution in each case. 2CD0 also contains a disulfide bond which appears to further stabilize its tertiary conformation. The loop bounded by SS bond-forming Cys residues conforms to the idealized distribution with good accuracy, suggesting a measure of coaction between both stabilizing factors (disulfides and hydrophobic interactions). It seems that the formation of the SS bond is enabled by hydrophobic forces which guide Cys residues towards the center of the protein body and into close proximity to each other. The resulting bonds do not, however, contribute to the formation of a shared (dimeric) hydrophobic core [54].

Of note is the variable status of β-folds: fragments 8–13, 19–26, 34–38, 45–48 and 69–76. Each of these folds contributes to the stability of either its parent monomer or the dimer.

Interface residues do not play an important role in structural stabilization of 2CD0 since eliminating them does not appreciably change the RD value.

**Figure 5:**
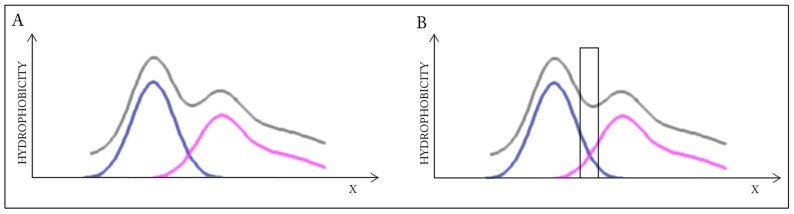
Schematic depiction of a system in which only one monomer contains a prominent hydrophobic core. Elimination of residues involved in complexation (right-hand image) leads to better accordance with the model when considering the dimer as a whole. Blue and pink lines: distributions in monomers, gray line: distribution in dimer. (**A**) Status of the individual monomers (**blue** and **pink**) versus the resultant distribution in dimer (**gray**); (**B**) The removal of the interface (**black bar**) does not change the status of the dimer—Distribution still discordant versus the expected one.

Under natural conditions, the V domain of the immunoglobulin light chain undergoes complexation with the corresponding domain of the heavy chain. Pathological conditions lead to formation of an excessive number of light chain dimers. The asymmetric nature of the homodimer (Table 5) may be a consequence of “replacing” the heavy chain with another copy of the light chain, producing a complex in which each monomer adopts a different conformation. The role of interface is schematically presented in Figure 5. Elimination of P-P residues further stabilizes the dimer but does not produce a shared core as defined by the fuzzy oil drop model (RD > 0.5).

The V domain dimer also plays an important role in amyloidogenesis research, given that products of the major human V kappa and V lambda gene families have been identified in AL deposits. One particular subgroup (lambda-6) has been found to be preferentially associated with this disease. Comparative analysis of the status of individual β-folds in immunoglobulin-like domains indicates that—Despite a high degree of structural similarity, they differ with respect to their adherence to the fuzzy oil drop model [55].

### 2.5. Complexes Stabilized through Formation of an Additional Shared Domain in the Interface Zone

An entirely unexpected form of interface is observed in the 1DXX and 1QAG homodimers. Here, fragments contributed by each monomer assemble to form a quasi-domain which is highly accordant with the idealized hydrophobicity distribution model and contributes to the protein’s biological activity. Note that both 1DXX and 1QAG are components of a cross-membrane link between the actin-based cytoskeleton of the muscle cell and the extracellular matrix—They must therefore adapt to external stimuli (e.g., stretching) and revert to their original conformation when such stimuli are not present.

### 2.6. Dystrophin (1DXX)

**Figure 6:**
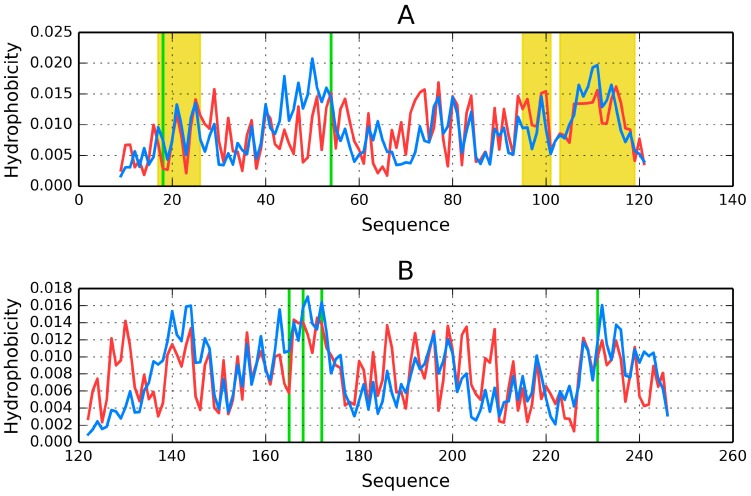
Theoretical (blue line) and observed (red line) hydrophobicity density distribution profiles in dystrophin: **A**—CH1 (complete domain), **B**—CH2 (complete domain). Green lines: positions of disease-related mutations, yellow area: fragment interacting with actin.

Figure 6 presents hydrophobicity density distribution profiles for complete domains CH1 and CH2 of dystrophin. Results (RD parameters) for the actin-binding N-terminal fragment of human dystrophin are listed in Table 6.

The “Modified” tag indicates domain structures identified on the basis of the FOD-based definition of a domain. “Modified” means deprived of the fragment of chain identified as participating in interface-domain. The leftmost column also lists mutations which cause Duchenne’s muscular dystrophy. The most detrimental mutations are underlined. Question marks indicate that a given mutation does not belong to the analyzed fragment, but is found in its immediate neighborhood.

In addition to secondary folds the table also lists residues involved in interaction with actin. The helix at 119–134 is referred to as “Helix X” in [22].

The four-chain complex (ABCD), taken as a whole, does not appear to include a hydrophobic core in the sense of the fuzzy oil drop model. Similar conditions are encountered in both sub-complexes (AB and CD), as well as in individual monomers (A, B, C and D). This strong discordance is caused by the presence of a cavity in the central part of the complex as well as in individual chains.

**Figure 7:**
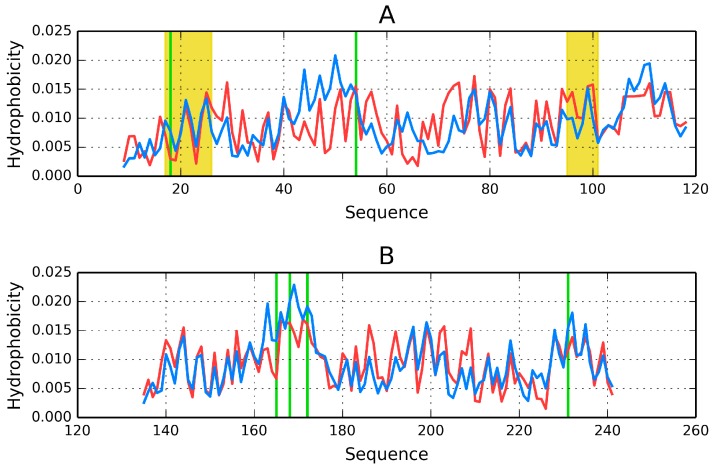
Theoretical (**blue line**) and observed (**red line**) hydrophobicity density distribution profiles. (**A**) CH1 (modified domain); (**B**) CH2 (modified domain). Green lines: positions of disease-related mutations. Yellow area: fragment interacting with actin.

The CH1 domain is highly accordant, unlike CH2, which does not contain a prominent core. The status of individual β-folds can be assessed on the basis of their RD coefficients, listed in Table 6. It appears that each domain, regardless of its overall status, includes locally discordant fragments. In CH1 the discordant fragment at 47–87 consists of two helixes joined by a twist. In CH2 the twist at 220–223 diverges from the model, likely causing the entire domain to slightly exceed the RD = 0.5 threshold (Figure 7).

Two fragments of CH1, which have been experimentally confirmed to mediate actin binding, retain accordance with the fuzzy oil drop model. It therefore seems likely that the act of complexation requires structural rearrangement of the entire CH1 domain as—Contrary to FOD predictions—No hydrophobic residues are exposed on the surface in the protein’s native form.

Visual inspection reveals fragments with high (e.g., the first 20 N-terminal residues and the 100–122 C-terminal segment in CH1) and low (e.g., 40–60 in CH1 and 120–140 in CH2) accordance between both distributions. In-depth comparative analysis is provided in Table 6.

**Figure 8:**
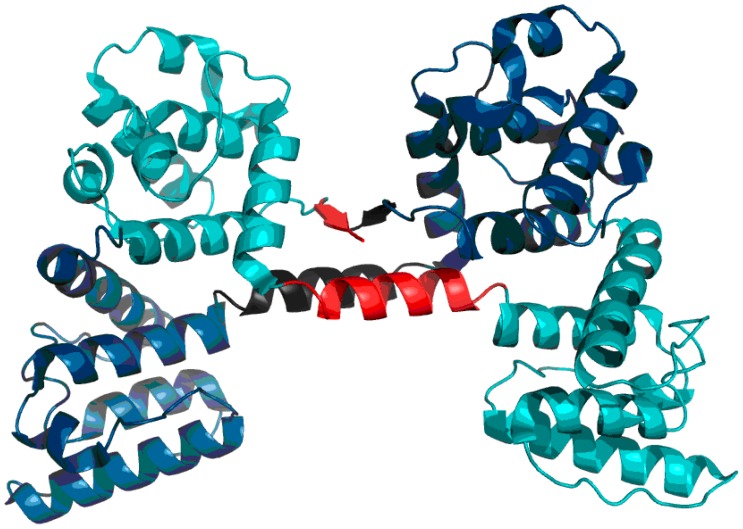
Structure of dystrophin: chain A (**turquoise**) with two fragments of the B chain (**dark blue**). The red fragments of the A chain interact with the corresponding black fragments of the B chain to produce the FOD domain.

**Figure 9:**
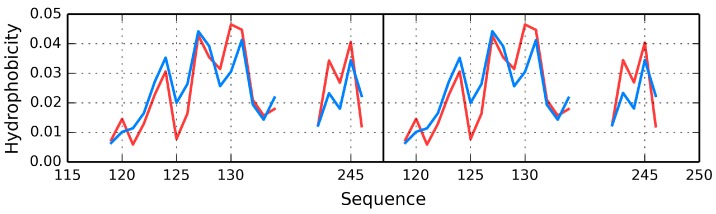
Hydrophobicity profile for the interface-domain in dystrophin comprising fragments of the A chain (**left**) (119–134 and 242–246) and analogous fragments of the B chain (**right**). Blue line: expected distribution; red line: observed distribution. The chart reveals good accordance between both profiles. Residues 119–134 form helixes, while residues 242–246 form β-folds.

Visual inspection of the structure of the AB complex (Figure 8) suggests a different division into domains than the one proposed in SCOP and PDBSum. In particular, an additional quasi-domain can be found sandwiched between CH1 and CH2. This domain is made up of fragments contributed by both chains. Modification of the set of primary domains does not appreciably alter the RD parameter of CH1; however it significantly improves the accordance of CH2 (Figure 8, Table 6). The quasi-domain, composed of fragments contributed by CH1 and CH2 in the AB interface zone, is also highly accordant, as suggested by T and O distribution profiles (Figure 9) and confirmed by low values of RD (Table 6 Refer to the “FOD domain” item). These values are 0.239 and 0.245 for the A/B and C/D complexes respectively.

**Figure 10:**
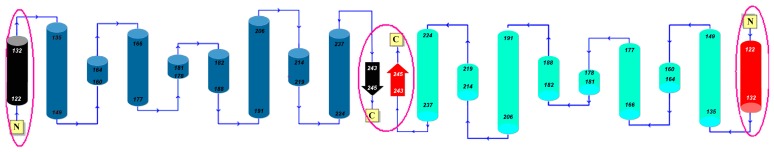
FOD domain structure distinguished by red ellipses. The color patter is identical to the one used in Figure 8.

As a conclusion, we propose a new type of domain consisting of fragments which belong to separate chains (Figure 10).

In Table 6 we also present mutations believed to cause Duchenne muscular dystrophy (DMD). The underlined items correspond to mutations which produce the most severe symptoms. Experimental studies indicate that all six mutant proteins are significantly more prone to thermal denaturation and aggregation [48]. It would be interesting to visualize the structure of mutant domains. Note that the most severe mutations affect a domain regarded as stable (under FOD criteria), although L54R is located in a fragment which exhibits high RD. It seems that any disruption in the complex interplay between stable and unstable fragments may severely impair the function of dystrophin, which (in its native form) needs to accommodate the stresses produced by stretching the membrane-actin link but nevertheless remain capable of reverting to its original conformation.

### 2.7. Utrophin

Much like dystrophin, the utrophin chain—When considered as a standalone unit—Diverges from the idealized hydrophobicity density distribution model. The chain itself resembles dystrophin in that it consists of two distinct domains; however unlike in dystrophin both domains conform to the FOD model.

In domain 1 (31–130 aa), the helical fragments at 63–75 and 85–103, as well as the adjoining loops (48–62 and 76–84) diverge from the model, whereas in domain 2 only the helix at 206–223 remains divergent. The inter-domain interface of utrophin differs from the one in dystrophin; only helical fragments are present and their spatial conformation is different. Nevertheless, calculations based on the FOD model indicate that the interface exhibits a hydrophobicity density distribution which is remarkably similar to its dystrophin counterpart and also in good agreement with the model (although dystrophin conforms to the model with greater accuracy).

**Figure 11:**
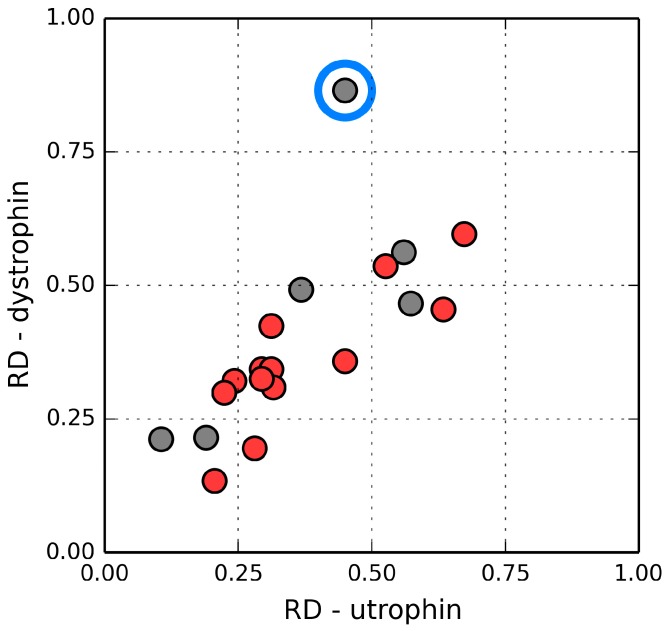
Relation between the status of individual secondary folds in dystrophin and in utrophin. The blue circle marks an outlier which corresponds to an exposed loop. Red: helices, gray: loops.

In general, the constituent domains of both proteins share similar FOD characteristics. Figure 11 illustrates the relations between their corresponding RD values. The correlation coefficient calculated for all analogous fragments is 0.715 (0.855 when excluding the single outlier, i.e., the loop at 235–238 in 1QAG and the corresponding loop at 220–223 in 1DXX—This loop is exposed on the surface and likely plays a role in interaction with the protein’s environment). The modified domain 2 (deprived of its N-terminal fragment) exhibits higher RD, but the value is still below 0.5. This suggests that the helix at 151–164 serves as a stabilizing connector for domain 2 as well as for the FOD quasi-domain.

**Figure 12:**
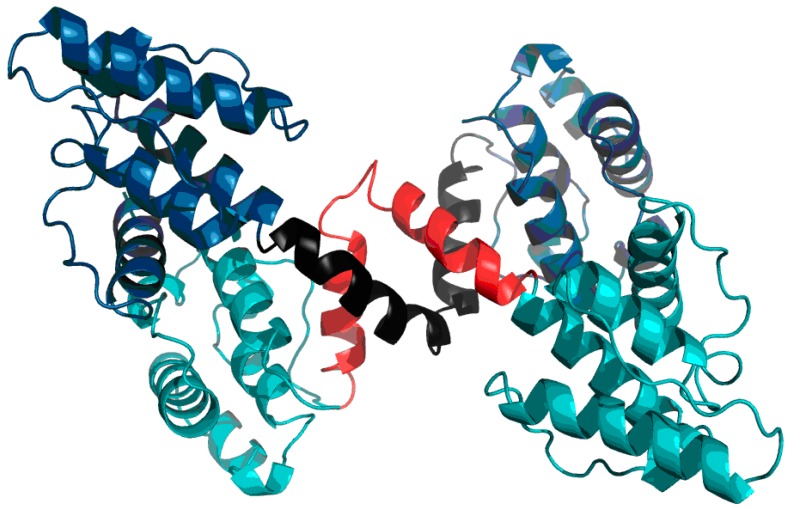
Utrophin homodimer (chain A: Dark blue; chain B: Teal). Fragments contributed to the domain interface by chains A and B are marked in black and red respectively.

Much like dystrophin, utrophin also contains a quasi-domain comprised of fragments contributed by individual chains (two helixes per chain). Figure 12 depicts these fragments (black fragment in chain A and red fragment in chain B. The resulting structure is hydrophobically stable, with an RD value of 0.453 (Table 7), indicating the presence of a well-defined hydrophobic core which stabilizes the complex.

**Figure 13:**
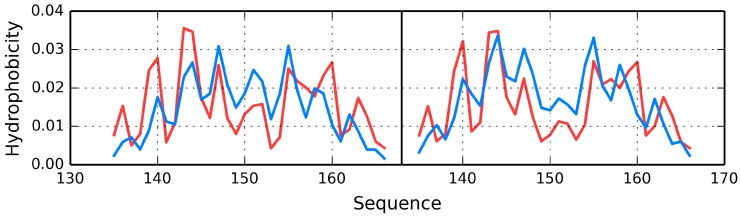
Utrophin-Theoretical (**blue line**) and observed (**red line**) hydrophobicity density profiles. The similarity between both distributions is evident.

The chart depicted in Figure 13 includes fragments contributed by chains A and B, revealing a highly coherent structure with its own hydrophobic core. This phenomenon suggests that the specific interplay of both chains exerts a stabilizing influence on the complex as a whole.

**Figure 14:**
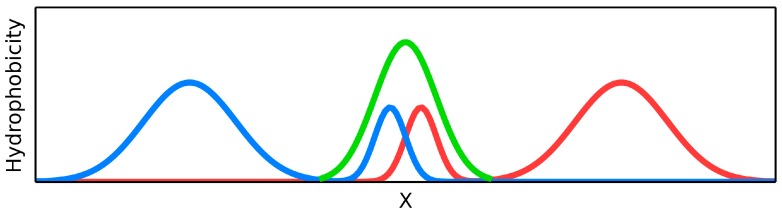
Schematic representation of the interface-domain formed by both chains in the interface area. Blue and red lines: ndividual chains, Green line: the additional domains generated by fragments of individual chains.

Figure 14 schematically depicts the presence of an additional quasi-domain in the interface zone. In this case, the quasi-domain is composed of inter-domain connectors (131–148) and the N-terminal fragment of domain 2 (149–166).

## 3. Discussion

The fuzzy oil drop model, which describes the hydrophobic core status in monomeric units (domains) and larger complexes (dimers), may assist researchers in the study of mechanisms responsible for quaternary structural stabilization. A dimeric complex may form around a shared hydrophobic core with a coherent distribution of hydrophobicity throughout the protein body. Alternatively, each monomer may retain its own hydrophobic core while interacting with its partner. An interesting phenomenon is observed in the case of dystrophin and utrophin: these proteins generate an independent quasi-domain in the interface zone, consisting of parts of both monomers and exhibiting very high accordance with the theoretical model. This solution appears to be correlated with the biological properties of both proteins: both dystrophin and utrophin bind actin filaments (N-terminal domains) with membrane glycoproteins (C-terminal sites) and must withstand substantial external forces, which produce structural deformations. Under these conditions structural stabilization by way of nonbinding interactions may prove insufficient, and a shared quasi-domain is needed to provide additional stability. This domain is not listed in protein domain databases since it is not entirely contained in either monomeric chain. It is also not recognized by CATH, which specifically focuses on individual polypeptide chains. Similarly, the C-terminal β-fold is not subject to formal classification as it lacks a suitable β-“partner” in the opposite chain (cf. secondary conformation scheme in PDBSum). In the case of utrophin and dystrophin the presented structural solution appears to be a consequence of these proteins’ biological properties: specifically, their susceptibility to stretching forces.

Comparative analysis of the dystrophin and utrophin conformations presented in [22] underscores the differing orientation (twisting angle) of domains CH1 and CH2, as well as differences in inter-domain interaction potential. The authors suggest that the dystrophin dimer is more stable of the two, and note that the utrophin dimer lacks a β-“latch” produced by the C-terminal fragment. From the point of view of the interface domain, the secondary conformation of individual folds is of relatively little importance—instead, the domain relies on the presence of a prominent hydrophobic core. Mutations which cause Duchenne or Becker’s muscular dystrophy do not seem to affect the analyzed interface domains; hence both diseases are likely unrelated to structural changes in this area [22].

A similar relation between structure and function can be observed in the immunoglobulin-like domain of titin (1TIT) [50,55]. This protein, which is expressed in muscle tissue, must adapt to external forces while retaining shape memory. Much like other proteins discussed in this paper, titin exhibits good accordance with the theoretical hydrophobicity density distribution model, with domain-specific RD values as low as 0.32. It seems that the presence of a well-ordered hydrophobic core is a critical factor in ensuring that the protein reverts to its original conformation in the absence of external forces (note, however, that other immunoglobulin-like domains which also follow the “two-layer sandwich” blueprint do not conform to the model with such high accuracy).

In summary, it should be noted that high concentrations of hydrophobicity density at the center of the domain exert a stabilizing influence on the domain as a whole. This observation is in agreement with the well-known relationship between hydrophobic interactions and tertiary structural stabilization.

The authors of [56] report that many digitally generated protein-protein interfaces with highly favorable computed binding energies could not be observed in practice. They conclude that there may be important physical chemistry missing in the energy calculations. The algorithm applied in [52,56] can be summarized as follows:

- Pre-compute a set of high-affinity amino acid residue interactions with the target surface;
- Redesign natural protein scaffolds to incorporate a selection of these amino acids;
- Design the remainder of the interface to enhance binding affinity.

We attempt to approach the presented issue by performing a holistic analysis of protein conformations on the basis of the fuzzy oil drop model.

Some other publications, which deal with changes triggered by point mutations in the structure of protein-protein interfaces, also highlight the need for improved energy functions [52].

Examples of structures characterized by variable FOD status can be found in the immunoglobulin-like domain family [55]. Despite their overall structural and sequential similarity, the conformance of individual folds which make up the β-sandwich varies from protein to protein.

This work attempts to underscore the influence of global (i.e., protein-wide) hydrophobicity density distribution upon the protein’s biological properties; particularly its ability to form complexes. It should be noted that earlier methods which focus entirely on pairwise atom-atom interactions (including interactions between protein atoms and the surrounding water environment) only acknowledge the effects of immediate “interaction partners”, while the structure of interfaces is critically dependent on the overall distribution of hydrophobicity density throughout the protein body, as well as on local deviations from the idealized model. The interface does not form in separation from other parts of the protein; rather, the entire molecule must participate in this process, folding in such a way as to ensure that the contact area remains capable of interacting with the intended ligand. This process relies on correct distribution of hydrophobicity density, including local discordances. Point mutations, not necessarily located close to the interface zone, may disrupt the protein’s hydrophobic core and lead to structural deformations which may render the protein less susceptible—Or indeed entirely incapable of—Complexation. In light of this phenomenon, existing in silico folding models, which acknowledge the presence of ligands or external proteins, should undergo further evolution [57].

## 4. Materials and Methods

### 4.1. Data

All structures were taken from PDB Data Base [58]. The identification of interface residues was performed according to PDBSum standards [51] using the distance between interacting atoms.

### 4.2. Programs

The 3D presentation of protein structures was generated using PyMol [59]. Calculations of RD values (complete calculation concerning FOD) were performed using our own software.

## Figures and Tables

**Table 1:** Complexes subject to analysis in the presented work. Columns indicate the status of the hydrophobic core in each monomer while rows correspond to the status of the shared (dimeric) core. Numbers in parentheses represent the number of monomers which exhibit accordance with the idealized hydrophobic core model. The rightmost column lists two proteins which contain an emergent quasi-domain, comprised of fragments contributed by each monomer and possessing its own hydrophobic core.

Dimer	Status	Monomer		Additional Interface Domain
		Hydrophobic Core		
		Present	Absent	
Hydrophobic core	present	1BFM [44]	1Y7Q [45]	
	absent	1R1V (2) [46]	2CD0 (1) [47]	
Additional interface domain				1DXX [22], 1QAG [26]

**Table 2:** RD coefficients describing the status of the hydrophobic core in the dimer and in each monomer of 1BFM. The table also lists the status (with respect to the monomer and the dimer) of individual helical folds. “No P-P” indicates parts of the monomers and the dimer following elimination of residues which make up the P-P interface.

1BFM	RD			
	Individual Monomer		Monomer in Dimer	
	A	B	A	B
Monomer	0.441	0.412	0.379	0.386
Helix				
4–16	0.429	0.355	0.360	0.379
21–51	**0.565**	**0.514**	0.480	0.457
56–68	0.260	0.201	0.315	0.300
NO P-P	0.452	0.365	0.390	
COMPLETE DIMER			0.383	

**Table 3:** RD coefficients describing the status of the hydrophobic core in the dimer and in each monomer of 1Y7Q. The table also lists the status (with respect to the monomer and the dimer) of individual helical folds. “No P-P” indicates parts of the monomers and the dimer following elimination of residues involved in protein-protein interactions.

1Y7Q	Individual Monomer		Monomer in Dimer	
	A	B	A	B
Monomer	**0.553**	**0.514**	0.410	0.430
Helix				
43–51	**0.571**	0.484	**0.522**	**0.521**
60–76	**0.563**	**0.558**	0.312	0.330
81–98	0.488	0.430	0.452	0.454
100–108	0.313	0.361	0.353	0.361
113–126	0.426	0.381	0.372	0.393
No P-P	**0.511**	0.478	0.471	
Complete Dimer			0.421	

**Table 4:** RD coefficients describing the status of the hydrophobic core in the dimer and in each monomer of 1R1V. Values given in bold are fragments of status expressed by RD > 0.5. H and B denote the secondary form of the particular fragment, helical and β-structural respectively. Position “No P-P” expresses the status of dimer with residues in interface eliminated form calculation.

1R1V	RD			
	Individual Monomer		Monomer in Dimer	
	A	B	A	B
Monomer	0.476	0.477	**0.639**	**0.638**
9–24 H	**0.595**	0.496	**0.695**	**0.716**
25–38 H	0.478	0.496	0.789	0.780
41–50 H	0.232	0.212	0.537	0.510
**52–66 H**	**0.544**	**0.543**	**0.510**	**0.504**
68–74 B	0.140	0.148	0.093	0.093
**77–83 B**	**0.542**	**0.575**	**0.548**	**0.591**
**84–100 H**	**0.538**	**0.534**	**0.528**	**0.536**
β-sheet	0.304	0.354	0.302	0.356
No P-P	0.467	0.465	0.477	
**Complete Dimer**			**0.638**	

**Table 5:** RD coefficients describing the status of the hydrophobic core in the dimer and in each monomer of 2CD0. The table also lists the status (with respect to the monomer and the dimer) of individual folds: helixes (H), β-folds (B), upper core, lower core fragments bounded by Cys residues forming SS bonds, as well as fragments following elimination of residues involved in protein-protein interaction.

2CD0	RD			
	Individual Monomer		Monomer in Dimer	
	A	B	A	B
	0.526	0.477	0.719	0.704
Secondary				
3–5 B	0.428	0.400	0.150	0.221
8–13 B	0.163	0.292	0.600	0.611
19–26 B	0.535	0.547	0.361	0.411
27–31 H	0.603	0.566	0.678	0.689
34–38 B	0.175	0.137	0.561	0.582
45–48 B	0.119	0.131	0.771	0.753
61–66 B	0.104	0.100	0.211	0.275
69–76 B	0.437	0.341	0.531	0.506
79–83 H	0.571	0.576	0.670	0.657
84–92 B	0.489	0.294	0.416	0.360
95–99 B	0.370	0.176	0.363	0.344
102–107 B	0.747	0.634	0.866	0.822
β-sheet-Upper core	0.359	0.321	0.376	0.398
β-sheet-Lower core	0.515	0.537	0.793	0.757
SS-bond loop	0.487	0.453	0.681	0.695
No P-P	0.516	0.446	0.686	
Complete Dimer			0.711	

**Table 6:** RD values calculated for the ABCD complex, for AB and CD subcomplexes, for a single chain and for individual domains (CH1, CH2) of dystrophin (1DXX), as well as for selected secondary structural folds. “FOD DOMAIN” represents fragments contributed by two chains (A,B) which form a coherent domain with its own FOD-compliant hydrophobic core.

Dystrophin1DXX		
	Chain Fragment	RD
Complex–Tetramer	**ABCD**	**0.815**
Complex–Dimer	**AB/CD**	**0.754/0.752**
Chain	**A/C**	**0.839/0.758**
Chain	**B/D**	**0.761/0.762**
Domain 1 CH1	9–121	0.458
CH1 Modified	9–118	0.462
Helix K18N	13–32	0.424
Actin binding	17–26	0.341
Loop	33–46	0.466
Helix L54R	**47–59**	**0.596**
Loop	**60–68**	**0.562**
Helix	**69–87**	**0.536**
Loop	88–94	0.212
Helix-Actin binding	95–101	0.299
Helix-Actin binding	103–119	0.343
Domain 2 CH2 Modified	**122–246** 135–241	**0.510** 0.336
	134–246	0.432
Helix	*135*–*149*	*0.309*
Loop	*150*–*159*	*0.215*
Helix*D165V*	160–164	0.358
Helix *D165V*, A168D, L172H	166–177	0.321
Helix	178–181	0.299
Helix	182–188	0.195
Helix	191–206	0.455
Loop	207–213	0.492
Helix	214–219	0.134
**Loop**	**220–223**	**0.865**
Helix Y231N	224–237	0.325
β	242–246	0.224
Fod Domain		
	A (Helix119-134 + A (β242–246)) + B (Helix119–134 + B (β242–246))	0.239
	C (Helix119-134 + C (β42–246)) + D (Helix119–134 + D (β242–246))	0.245

**Table 7:** RD values calculated for the AB complex, for chains A and B and for individual domains of utrophin (1QAG), as well as for selected secondary structural folds. “Fod Domain” represents fragments contributed by two chains (A,B) which form a coherent quasi-domain with its own FOD-compliant hydrophobic core.

Utrophin 1QAG		RD
Complex	**AB**	**0.781**
Chain	**A/B**	**0.738/0.738**
Domain 1	31–130	0.493
Helix	31–47	0.312
Loop	**48–62**	**0.573**
Helix	**63–75**	**0.673**
Loop	**76–84**	**0.560**
Helix	**85–103**	**0.526**
Loop	104–110	0.106
Helix	111–118	0.294
Helix	119–130	0.312
Domain 2	149–251	0.390
Domain Modified	167–251	0.427
Helix	151–164	0.316
Loop	165–175	0.190
Helix	176–180	0.450
Helix	182–192	0.243
Helix	193–197	0.224
Helix	198–205	0.281
Helix	206–223	0.634
Loop	224–228	0.368
Helix	229–234	0.206
Loop	235–238	0.450
Helix	239–251	0.294
Fod Domain		
	A(134–166) + B(134–166)	0.453
